# What Drives Students to Pursue Gerontology and Geriatrics Education?

**DOI:** 10.1093/geroni/igaf122.3489

**Published:** 2025-12-31

**Authors:** Isaac Asirifi Boateng, Rona Karasik

**Affiliations:** St. Cloud State University, St. Cloud, Minnesota, United States; Saint Cloud State University, Saint Cloud, Minnesota, United States

## Abstract

Despite the growing need for practitioners, researchers and policymakers knowledgeable about aging and older adults, student interest in pursuing gerontology and geriatrics at all levels (e.g., undergraduate, graduate, post-graduate) and related fields (e.g., human services, medicine, PT/OT, social work) is failing to keep pace with demand. While student recruitment concerns in gerontology are not new, the situation is becoming increasingly dire. Previous studies have shown students are reluctant to enter the field because of ageism/negative attitudes toward aging and older adults (e.g., Jester et al., 2021), lack of awareness of career options (e.g., Dassel et al, 2014) and expectations of lesser prestige and compensation (e.g., Cheung et al., 2023). There is limited understanding, however, of what does draw potential students to gerontology and geriatrics. To develop effective strategies for increasing student interest in gerontological education, this study explored the motivations of individuals who chose the field. Findings from the first wave of data obtained through an email survey of current and recently graduated students taking courses in aging (N = 67) revealed several factors leading to their decisions, including: personal experiences with aging family members (n = 43), desire to improve quality of life for older adults (n = 39), policy advocacy (n = 24), ability to educate others on aging issues (n = 23), positive academic influences (n = 18), and aging-related service experiences (n = 18). Implications for these preliminary findings include opportunities for fostering greater interest in the field, enhancing an age-savvy workforce, and advocating for policies to support the growth of academic gerontology programs.

